# Supplementary figures and images for: Does the combination of exercise and cognitive training improve working memory in older adults? A systematic review and meta-analysis
Source: PeerJ. 2023 Apr 10;11:e15108. doi: 10.7717/peerj.15108 (PMC10100799; doi:10.7717/peerj.15108)

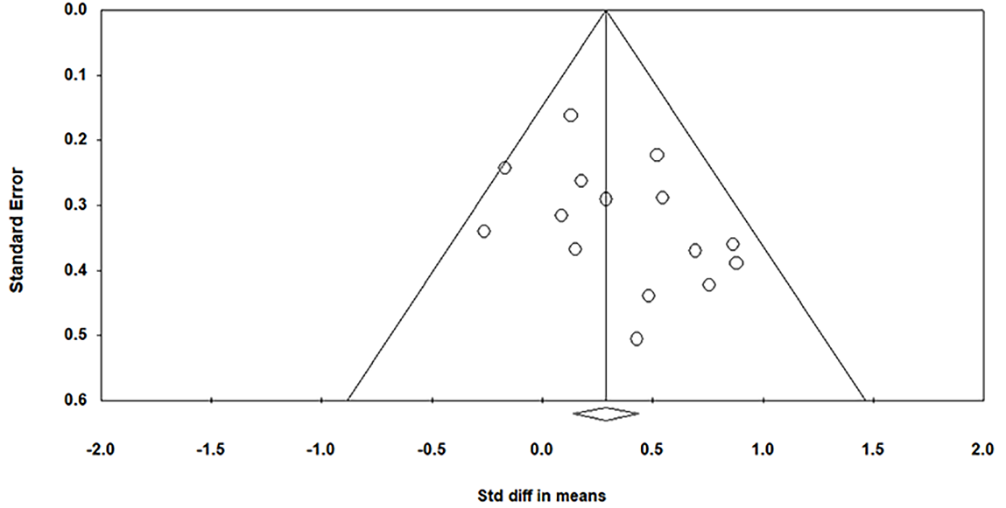

Supplement: Supplemental Information 2 [file peerj-11-15108-s002.png]

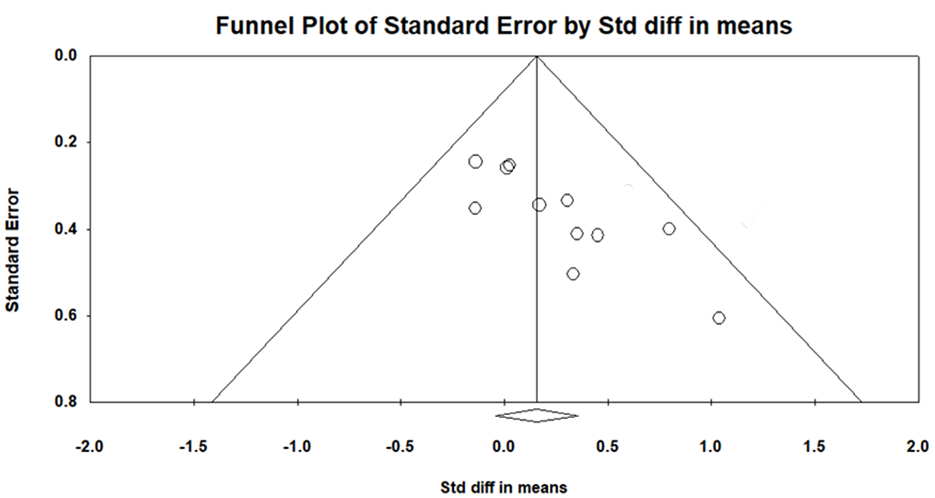

Supplement: Supplemental Information 3 [file peerj-11-15108-s003.png]

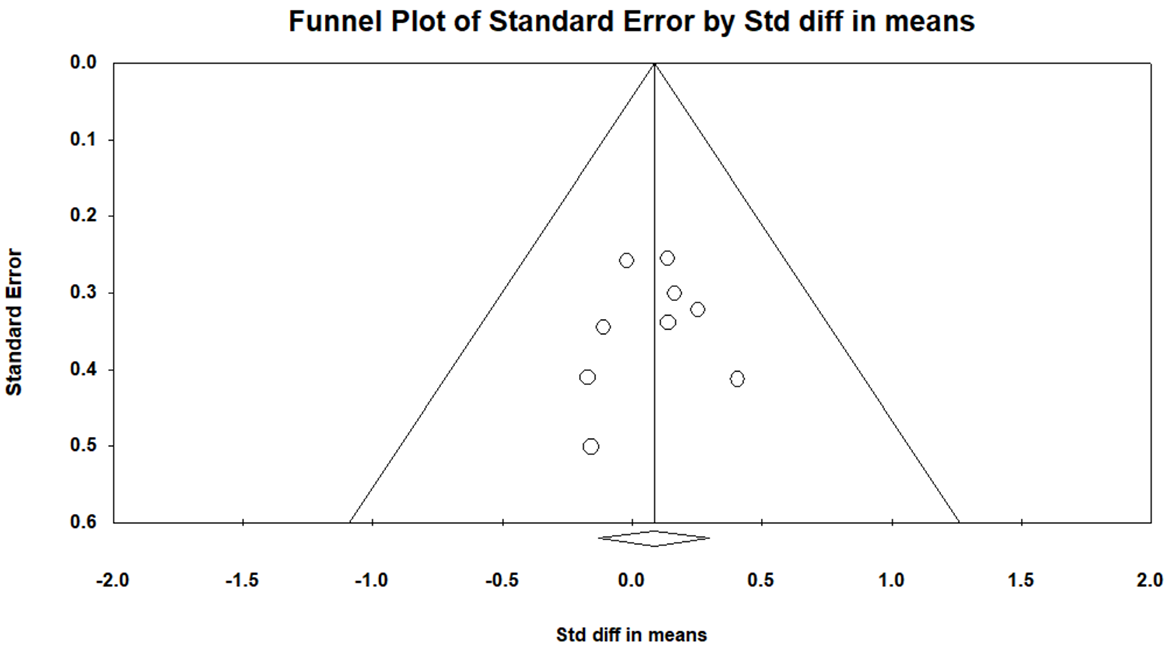

Supplement: Supplemental Information 4 [file peerj-11-15108-s004.png]
