# Supplementary material for: Does the combination of exercise and cognitive training improve working memory in older adults? A systematic review and meta-analysis
Source: PeerJ. 2023 Apr 10;11:e15108. doi: 10.7717/peerj.15108 (PMC10100799; doi:10.7717/peerj.15108)
Supplement: Supplemental Information 5 [file peerj-11-15108-s005.docx]

This meta-analysis uses the PEDro scale to assess study quality. When the study meets a criterion in the scale, they will get one point. The quality score for each article ranged from 0 to 11. The scores results are as follows:

Table S1. The quality of Included Studies

| **Study** | **Quality Assessment Criteria** | | | | | | | | | | | **Score** |
| --- | --- | --- | --- | --- | --- | --- | --- | --- | --- | --- | --- | --- |
|  | 1 | 2 | 3 | 4 | 5 | 6 | 7 | 8 | 9 | 10 | 11 |  |
| Fabre  (2002) | √ | √ | - | √ | - | - | - | √ | √ | √ | √ | **7** |
| You  (2009) | √ | √ | - | √ | - | - | - | × | √ | √ | √ | **6** |
| Legault  (2011) | √ | √ | - | √ | √ | - | - | √ | √ | √ | √ | **8** |
| Maillot  (2012) | √ | √ | - | √ | - | - | - | √ | √ | √ | √ | **7** |
| Suzuki  (2012) | √ | √ | - | √ | - | - | - | √ | √ | √ | √ | **7** |
| Shatil  (2013) | √ | √ | - | √ | - | - | - | × | √ | √ | √ | **6** |
| Nishiguchi (2015) | √ | √ | - | √ | × | √ | - | √ | √ | √ | √ | **8** |
| Rahe  (2015) | √ | √ | - | √ | × | - | √ | √ | √ | √ | √ | **8** |
| Gschwind  (2015) | √ | √ | - | √ | × | - | √ | √ | √ | √ | √ | **8** |
| Eggenberger  (2016) | √ | √ | - | √ | √ | × | × | √ | √ | √ | √ | **8** |
| Schättin  (2016) | √ | √ | - | √ | √ | × | × | √ | √ | √ | √ | **8** |
| Damirchi  (2017) | √ | √ | - | √ | - | - | - | √ | √ | √ | √ | **7** |
| Ordnung  (2017) | √ | √ | - | √ | - | - | - | √ | √ | √ | √ | **7** |
| Kalbe  (2018) | √ | √ | - | √ | × | - | √ | √ | √ | √ | √ | **8** |
| Donnezan  (2018) | √ | √ | - | √ | - | - | - | √ | √ | √ | √ | **7** |
| Bae  (2019) | √ | √ | - | √ | × | √ | - | √ | √ | √ | √ | **8** |
| Norouzi  (2019) | √ | √ | - | √ | × | - | - | √ | √ | √ | √ | **7** |
| Karssemeijer （2019） | √ | √ | - | √ | × | - | √ | √ | √ | √ | √ | **8** |
| Dana  (2019) | √ | √ | - | √ | - | - |  | √ | √ | √ | √ | **7** |
| Adcock  (2020) | √ | √ | √ | √ | × | × | × | √ | √ | √ | √ | **8** |
| Takeuchi  (2020) | √ | √ | - | √ | - | - | √ | √ | √ | √ | √ | **8** |
