# Supplementary material for: Does the combination of exercise and cognitive training improve working memory in older adults? A systematic review and meta-analysis
Source: PeerJ. 2023 Apr 10;11:e15108. doi: 10.7717/peerj.15108 (PMC10100799; doi:10.7717/peerj.15108)
Supplement: Supplemental Information 6 [file peerj-11-15108-s006.docx]

Table S2. Inclusion and Exclusion Criteria of Included Studies

|  | Inclusion criteria | Exclusion criteria |
| --- | --- | --- |
| Population | participants were elderly (aged 60 and over) and included healthy individuals along with those with MCI and dementia | participants aged < 60 years old |
| Intervention | combination of exercise and cognitive intervention | without combination of exercise and cognitive intervention |
| Comparison | at least one comparison group (combined intervention group with cognitive intervention alone or physical exercise intervention alone or a control group receiving no intervention) | studies that did not compare the combined intervention group with other intervention groups alone |
| Outcome | measurements of working memory included at least one outcome that could be used to calculate an effect size | studies without relevant data on working memory pre and post intervention |
| Study design | randomized controlled trial (RCT) design | no RCT design |
| Others | written in English | noninterventional studies; reviews and theoretical articles; case and protocol articles; unpublished studies and papers;  no written in English |
